# Immune checkpoint inhibitors for advanced non-small cell lung cancer: emerging sequencing for new treatment targets

**DOI:** 10.1136/esmoopen-2017-000200

**Published:** 2017-07-29

**Authors:** Pedro Nazareth Aguiar, Ramon Andrade De Mello, Carmelia Maria Noia Barreto, Luke Alastair Perry, Jahan Penny-Dimri, Hakaru Tadokoro, Gilberto de Lima Lopes

**Affiliations:** 1 PhD Student Programme, Faculty of Medicine of ABC, Santo André, SP, Brazil; 2 Department of Biomedical Sciences and Medicine, Division of Medical Oncology, University of Algarve, Faro, Portugal; 3 Division of Medical Oncology, Federal University of São Paulo, São Paulo, Brazil; 4 Monash University, Victoria, Australia; 5 Sylvester Comprehensive Cancer Center, University of Miami, Miami, Florida, USA

**Keywords:** immunotherapy, non-small cell lung cancer, pembrolizumab, nivolumab, atezolizumab

## Abstract

Lung cancer is the leading cause of cancer-related deaths in the world. Immune checkpoint inhibitors (ICI) stimulate cytotoxic lymphocyte activity against tumour cells. These agents are available for the treatment of non-small cell lung cancer (NSCLC) after failure of platinum-based therapy. One recent study has demonstrated that ICI monotherapy was superior to platinum-based chemotherapy for first-line treatment. Nevertheless, this benefit was only for a minority of the population (30%) whose tumour programmed death receptor ligand-1 (PD-L1) expression was above 50%. Therefore, several strategies are under investigation. One option for patients with PD-L1 expression lower than 50% may be the combination of ICI with platinum-based chemotherapy or with ICIs against different targets. However, all of these combinations are at an early stage of investigation and may be very expensive or toxic, producing several harmful adverse events.

## Lung cancer epidemiology

Lung cancer is the most common malignancy worldwide with more than 1.8 million new cases diagnosed in 2012.^1^ Lung cancer mortality is also high; North-American epidemiological data showed that the incidence and mortality rates of lung cancer in the USA was 57.3 and 46.0 per 100 000, respectively, with 224 390 new cases and 158 050 deaths estimated in 2016.^2 3^ As a result, lung cancer is the most common cause of cancer-related deaths in the world with more than 1.5 million deaths worldwide in 2012.^1 3^ Recent trends suggest that in the USA overall lung cancer mortality rates are decreasing, but for women reduction in mortality is occurring at a disparately lower rate than men, probably because of smoking habits.^4^

Non-small cell lung cancer (NSCLC) is a histological class comprising approximately 85% of all lung cancers and at diagnosis a majority of patients with NSCLC have metastatic disease.^3 5^

## Treatment of metastatic NSCLC

In the past, the outcome of treatment for patients with advanced NSCLC was poor, with a median survival of 4–5 months and a 1-year survival rate of 10%.^6^ Platinum-based therapies as well as non-platinum based single agents like paclitaxel, docetaxel, gemcitabine, vinorelbine and irinotecan have improved the median survival to 7–9 months and 1-year survival rate to over 35%.^7^ Thereafter, trials comparing single-agent cisplatin with cisplatin in combination with newer agents have showed significant improvement in overall survival (OS) with combination therapy.^8 9^ Among combinations under investigation, none proved superior to the others.^10^

More recently, agents like pemetrexed and bevacizumab combined with platinum-based therapies for four to six cycles and single-agent or combination maintenance until disease progression have further improved survival.^11–14^ However, both drugs are approved only for patients with non-squamous histology (non-squamous cell carcinoma). Pemetrexed has been shown to decrease the survival rate among squamous histology (squamous cell carcinoma (SCC)),^11^ and bevacizumab had a higher risk of haemoptysis for SCC as previously observed in phase II studies.^12^

Despite all these improvements, the median progression-free survival (PFS) observed with chemotherapy is around 6 months and the median OS do not surpass 15 months in most trials of non-targeted agents.^13 14^

In the last decade, better understanding of molecular pathways led to the development of targeted therapies and personalised medicine in lung cancer. The human epidermal growth factor receptor (EGFR) is the most studied target in lung cancer. This is a transmembrane receptor with an extracellular portion containing a binding domain for growth factors and an intracellular domain including tyrosine kinase that through the RAS/RAF/MEK/ERK pathway leads cell proliferation, angiogenesis and cellular immortality.^15^ Mutations of the gene encoding EGFR are present in 15% to 60% of adenocarcinomas, and are related to Asian ethnicity, female gender and absence of smoking.^16^

When mutated, the receptor remains active and cellular proliferation signals become continuous and disinhibited. Treatment with tyrosine kinases inhibitors (TKIs) lead to tumour responses in over 50% of patients and nearly doubles median PFS.^16–18^ Another key oncogenic mutation has been discovered and studied: the anaplastic lymphoma kinase (ALK) gene. ALK is rearranged in up to 6% of patients with NSCLC.^15^ ALK-targeted TKIs have been developed and show similar efficacy as EGFR TKIs.^19^

After progression on first-line platinum-based combination therapy or targeted agents, there are only a few treatment options available. Docetaxel, pemetrexed and erlotinib^20^ achieve a median PFS between 2 and 3 months and median OS of around 8 months, underscoring the need for more treatment options.^20^

## NSCLC and the immune system

Tumour cells (TCs) acquire several mutations during their development. These mutations may lead to TC immortality and aberrant proliferation. However, some of these mutations can produce aberrant proteins that can serve as neoepitopes, which are recognised by the immune system.^21^ Not all tumours have the same burden of mutations, and it is believed that a higher tumour burden leads to higher immunogenicity.^21^ Squamous and non-squamous NSCLCs, as well as melanoma, have the highest burden of mutations, and they were among the first studied in the development of immunotherapy.^22^

The immune system is able to recognise and destroy TCs as well as pathogenic agents. Nevertheless, one of the hallmarks of cancer is its ability to evade the immune system.^23^ There are many complex interactions between antigen presenting cells, lymphocytes and TCs. The most studied is the link between the lymphocytic membrane receptor, programmed cell death 1 (PD-1) and its ligands 1 or 2 (PD-L1 or PD-L2), which are often expressed by TCs. The interaction between PD-1 and PD-L1/PD-L2 inhibits lymphocytes and stimulates their apoptosis.^21^

Immune checkpoint inhibitors (ICIs) are an emerging class of immunotherapy which stimulate lymphocytes against TCs and might be better tolerated than cytotoxic chemotherapy. The most studied class in the treatment of NSCLC is anti-PD1/anti-PD-L1 drugs.^21^

## Immune checkpoint inhibitors for second-line treatment of NSCLC: phase I/II trial data

Nivolumab is a fully human monoclonal antibody against PD-1. It was the first immune checkpoint inhibitor approved for second-line treatment of NSCLC. CheckMate-003 was a phase Ib clinical trial that assessed nivolumab at 1, 3 or 10 mg/kg every 2 weeks in 129 heavily pretreated patients with advanced NSCLC regardless of tumour histology.^24^ The median OS was 9.9 months, and the 1-year survival rate was 42%.^24^ The objective response rate (ORR) was 17%.^24^ Nivolumab was well tolerated (14% of patients had grade 3 or 4 adverse events);however there were three fatal cases of pneumonitis.^24^ These results generated significant enthusiasm for further development of lung cancer immunotherapy.

CheckMate-063, a phase II clinical trial, evaluated the efficacy and safety of nivolumab at 3 mg/kg every 2 weeks for 117 patients previously treated for squamous NSCLC.^25^ The 1-year survival rate was 40.8%, and the median OS was 8.2 months; 14.5% of patients had a partial response.^25^ There were four non-fatal cases of grade 3 pneumonitis and little severe toxicity (17% of patients had grade 3 or 4 adverse events).^25^ Three-quarters of patients had their tumour sample assessed for PD-L1 expression and the cut-off value for positivity was considered to be 5%. The ORR was higher for patients with PD-L1 above the cut-off than those below (24% and 14%, respectively).^24^

Pembrolizumab is a humanised monoclonal antibody that targets the PD-1 receptor. KeyNote-001 was a large phase Ib trial that included 495 patients with advanced NSCLC (80% of whom were previously treated) who received pembrolizumab at 2 or 10 mg/kg every 3 weeks or 10 mg/kg every 2 weeks.^26^ The median OS was 12 months for all patients (9.3 months for previously treated patients and 16.2 months for previously untreated patients); 19.4% of all patients had partial responses.^26^ All patients had their tumour samples assessed for PD-L1 expression and the ORR was directly proportional to PD-L1 expression (8.1% for PD-L1<1%, 12.9% for PD-L1 1%–24%, 19.4% for PD-L1 25%–49%, 29.6% for PD-L1 50%–74% and 45.4% for PD-L1≥75%).^26^ This study also reported that patients who expressed PD-L1 had higher survival rates than those who did not: the median OS was around 9 months for patients with PD-L1<1% or PD-11%–49% and the median OS was not reached for patients with PD-L1≥50%.^26^

Atezolizumab is a humanised monoclonal antibody that acts in the same pathway as nivolumab and pembrolizumab, but its target is PD-L1. This monoclonal antibody was studied in a dose escalation phase Ia trial that included 88 patients with advanced NSCLC (11% of them previously untreated).^27^ The ORR and median OS for all patients were 23% and 16 months, respectively.^27^ In this study, PD-L1 expression ≥50% in TCs or tumour-infiltrating cells resulted in higher ORR (48% vs 16%) and a longer median OS (18 months vs 16 months).^27^ Atezolizumab was well tolerated with 11% of patients experiencing at least one grade 3 to 4 adverse event; there were four cases of pneumonitis, but none was severe.^27^

Avelumab is a fully human monoclonal antibody against PD-L1. It was studied at a dose of 10 mg/kg every 2 weeks in a large phase Ib trial that included 184 patients with previously treated NSCLC.^28^ The ORR with avelumab was 13.6%, the median OS 8.4 months and the 1-year survival rate was 37%.^28^ A majority of patients had their tumour samples assessed for PD-L1 expression (142) based on a cut-off of ≥1% TCs with staining of any intensity.^28^ Although differences were not statistically significant, PD-L1 positive patients had higher ORR (15.6% vs 10%) and longer median OS (8.9 months vs 4.6 months).^28^ The safety profile was compatible with previously described results with 12.5% of patients experiencing adverse events of grade 3 to 4, including two cases (1.1%) of pneumonitis, one of which was fatal.^28^

Durvalumab is a fully human IgG1 antibody against PD-L1. The drug was studied in a dose escalation trial that included 228 patients with advanced NSCLC (12% of them previously untreated).^29^ The ORR for all patients was 16% (15% for patients previously treated) and the median OS was 8.9 months for PD-L1-negative patients and the median OS was not reached for PD-L1-positive patients.^29^ The ORR was also associated with PD-L1 expression (27% for PD-L1-positive patients and 5% for PD-L1-negative patients).^29^ Durvalumab was well tolerated; there were only three non-fatal cases of pneumonitis and only 8% of the patients had adverse event of grade 3 to 4—table 1).

**Table 1:** Summary of results from non-randomised clinical trials

Study	Drug	Population	n	ORR	OS
CheckMate-003^24^	Nivolumab	NSCLC previously treated	129	17%	9.9 months
CheckMate-063^25^	Nivolumab	SCC previously Treated	117	14.5%	8.2 months
KeyNote-001^26^	Pembrolizumab	NSCLC 80% previously treated	495	19.4%	12 months
PCD4989 g^27^	Atezolizumab	NSCLC 89% previously treated	88	23%	16 months
Javelin^28^	Avelumab	NSCLC 99% previously treated	184	13.6%	8.4 months
Rizvi^29^	Durvalumab	NSCLC 88% previously treated	228	16%	8.9 months (PD-L1 −) NR (PD-L1 +)

NSCLC, non-small cell lung cancer; ORR, objective response rate; OS, overall survival; PD-L1, programmed death receptor ligand-1.

## Immune checkpoint inhibitors for the second-line treatment of NSCLC: randomised clinical trials

CheckMate-017 evaluated nivolumab 3 mg/kg every 2 weeks versus docetaxel 75 mg/m² every 3 weeks for the second-line treatment of patients with squamous NSCLC.^30^ The study randomised 272 patients (135 for nivolumab and 137 for docetaxel).^30^ Nivolumab showed a statistically significant benefit in ORR (20% vs 9%; p=0.008), PFS(median 3.5 months vs 2.8 months; HR 0.62, 95% CI 0.47 to 0.81; p<0.001), and OS (median 9.2 months vs 6 months; HR 0.59, 95% CI 0.44 to 0.79; p<0.001).^30^ Nivolumab was better tolerated than docetaxel; treatment-related adverse events leading to discontinuation were less frequent in the nivolumab group (3% vs 10% of patients).^30^ Myelotoxicity occurred in up to 30% of patients treated with docetaxel and was a rare event (1% to 2% of patients) with nivolumab.^30^ Pneumonitis occurred in six cases with nivolumab, but none was severe.^30^ Furthermore, there were no deaths related to the treatment in the nivolumab group, and three deaths related to therapy in the docetaxel arm.^30^

CheckMate-057 compared nivolumab 3 mg/kg every 2 weeks with docetaxel 75 mg/m² every 3 weeks in patients with advanced non-squamous NSCLC.^31^ Five hundred and eighty-two patients were randomly assigned to nivolumab (292 patients) or docetaxel (290 patients).^31^ As in CheckMate-017, nivolumab was better tolerated (5% of patients discontinued nivolumab due to adverse events versus 15% of those treated with docetaxel) and was associated with increased ORR compared with docetaxel (19% vs 12%; p=0.02).^31^ There was no reported benefit in PFS and this may be due to the atypical pattern of response observed with immunotherapy (such as pseudoprogression).^32^ Nivolumab was associated with increased median OS (12.2 months vs 9.4 months; HR 0.72, 95% CI 0.60 to 0.88; p<0.001) when compared with docetaxel.^31^

Both studies retrospectively evaluated PD-L1 expression as a predictive biomarker. PD-L1-positive patients had better ORR and OS compared with PD-L1-negative patients.^30 31^ Interestingly, the correlation between PD-L1 expression and clinical benefits, such as ORR, PFS and OS, were more significant for patients with non-squamous tumours.^30 31^

KeyNote-010 was a phase IIb/III trial evaluating the efficacy of pembrolizumab versus docetaxel in patients with previously treated advanced NSCLC with PD-L1 expression of at least 1%.^33^ The authors screened 2222 patients’ tumour samples for PD-L1 expression and found 1475 (66%) with at least 1% expression.^33^ Investigators randomised 1034 patients in a 1:1:1 ratio to receive pembrolizumab 2 mg/kg every 3 weeks, pembrolizumab 10 mg/kg every 3 weeks or docetaxel 75 mg/m² every 3 weeks.^33^ Pembrolizumab improved the median OS compared with docetaxel (pembrolizumab 2 mg/kg vs docetaxel: 10.4 months vs 8 months; HR 0.71, 95% CI 0.58 to 0.88; p=0.0008; pembrolizumab 10 mg/kg vs docetaxel: 12.7 months vs 8 months; HR 0.61, 95% CI 0.49 to 0.75; p<0.0001).^33^ The ORR was also improved (18% for patients treated with pembrolizumab 2 or 10 mg/kg and 9% for patients treated with docetaxel).^33^ The benefits with pembrolizumab were more pronounced among patients with PD-L1 expression ≥50% (HR for OS was 0.50, 95% CI 0.36 to 0.70; p<0.0001).^33^ Severe adverse events were less common for patients treated with pembrolizumab (13% for pembrolizumab 2 mg/kg, 16% for pembrolizumab 10 mg/kg, and 35% for docetaxel).^33^ The most common side effects in patients treated with pembrolizumab were decreased appetite, fatigue, rash and nausea.^33^

POPLAR was a multicentre, randomised, phase II trial comparing atezolizumab 1200 mg every 3 weeks versus docetaxel 75 mg/m² every 3 weeks for patients with NSCLC who progressed on platinum-doublet chemotherapy.^34^ Two hundred and eighty-seven patients were included in this study: 144 were randomly allocated to receive atezolizumab and 143 to the docetaxel group.^34^ Atezolizumab improved median OS (12.6 months for atezolizumab vs 9.7 months for docetaxel), although the ORR was not improved in the overall population (15% for both groups).^34^ All patients had their tumour samples assessed for PD-L1 expression and the researchers evaluated TCs as well as tumour-infiltrating cells.^34^ The ORR with atezolizumab was directly proportional to the PD-L1 expression (8% for patients with PD-L1 negative and 38% for patients with PD-L1≥50%).^34^ In the atezolizumab arm, not only was OS higher among PD-L1-positive patients but this subgroup were the only group with a statistically significant improvement compared with docetaxel.^34^ OS HR for all patients was 0.77 (95% CI 0.55 to 1.06); HR for PD-L1≥50% was 0.46 (95% CI 0.19 to 1.09)—median OS not reached with atezolizumab; HR for PD-L1-negative patients was 1.12 (95% CI 0.64 to 1.93)—median OS 9.7 months with atezolizumab.^34^

The OAK tial evaluated atezolizumab for the second-line treatment of NSCLC regardless of tumour histology or PD-L1 expression; however, there was a stratification according to PD-L1 expression.^35^ Investigators enrolled 1225 patients and randomised them to atezolizumab (1200 mg every 3 weeks) or docetaxel (75 mg/m² every 3 weeks).^35^

In the preliminary analysis of data from 850 patients (425 included in each treatment arm), the OS improved by 27% in the patients receiving atezolizumab compared with those treated with docetaxel (median OS was 13.8 months vs 9.6 months; HR 0.73; 95% CI 0.62 to 0.87).^35^ There was no improvement in ORR (14% for atezolizumab vs 13% for docetaxel).^35^

When patients were stratified according to their level of PD-L1 expression, the OS was 59% greater among patients with PD-L1 expression ≥50% or≥10% in the infiltrating cells who were treated with atezolizumab, compared with the same group treated with docetaxel.^35^ The median OS was 20.5 months for atezolizumab and 8.9 months for docetaxel (HR 0.41; 95% CI 0.27 to 0.64).^35^ However, even inpatients without PD-L1 expression, there was a significant improvement in OS with atezolizumab compared with docetaxel.^35^ Among PD-L1-negative patients, the median OS was 12.6 months for atezolizumab versus 8.9 months for docetaxel (HR 0.75; 95% CI 0.59 to 0.96).^35^

PD-1 blockade can enhance lymphocyte function in a diversity of organs and systems while PD-L1 blockade may stimulate lymphocytes only in the tumour microenvironment because PD-L1 is much more common in TCs than normal cells.^21^ Although many scientists expected a more favourable toxicity profile because of high specificity of PD-L1 blockade, atezolizumab showed a similar adverse events profile to those previously reported for anti-PD-1 drugs.^35^

Curiously, an anti-PD-L1 treatment was the first immune checkpoint inhibitor that showed statistically significant OS improvement among PD-L1-negative patients. In the literature, this phenomenon remains unexplained. An often quoted hypothesis is that differences in PD-L1 testing assay used in each study might explain these different results.^36^ The specific SP142 monoclonal antibody used in the trials with atezolizumab seems to be less sensitive than other monoclonal antibodies such as 22C3.

All randomised clinical trial that included patients with both squamous and non-squamous histology found improvements in OS similar in both histology subtypes.^33–35^ Furthermore, all randomised clinical trial showed that patients with EGFR mutations had lower benefits with immunotherapy compared with patients who were EGFR wild type (table 2).^31 33–35^

**Table 2:** Randomised clinical trial data

	Immunotherapy versus docetaxel	ORR versus docetaxel	Median PFS versus	Median OS versus docetaxel	HR for OS (95% CI)
CheckMate-017^30^	Nivolumab	20% vs 9%	3.5 m vs 2.8 m	9.2 m vs 6.0 m	0.59 (0.44 to 0.79)
CheckMate-057^31^	Nivolumab	19% vs 12%	2.3 m vs 4.2 m	12.2 m vs 9.4 m	0.73 (0.59 to 0.89)
KeyNote-010^33^	Pembrolizumab 2 mg/kg	18% vs 9%	3.9 m vs 4.0 m	10.4 m vs 8.5 m	0.71 (0.58 to 0.88)
	Pembrolizumab 10 mg/kg	18% vs 9%	4.0 m vs 4.0 m	12.7 m vs 8.5 m	0.61 (0.49 to 0.75)
POPLAR^34^	Atezolizumab	15% vs 15%	2.7 m vs 3.0 m	12.6 m vs 9.7 m	0.73 (0.53 to 0.99)
OAK^35^	Atezolizumab	14% vs 13%	2.8 m vs 4.0 m	13.8 m vs 9.6 m	0.73 (0.62 to 0.87)

ORR, objective response rate; OS, overall survival; PFS, progression-free survival.

## The Tale of the Tail

The main issue to support immunotherapy is the existence of a proportion of patients who will reach a life-time long benefit with the treatment. This is ‘The Tale of the Tail’ that had been observed previously in the treatment of melanoma.^37^ However, new data indicate that the same phenomenon can also occur in the second-line treatment of NSCLC.

The first evidence of this derives from the data of 2-year follow-up of previously published randomised clinical trials. Data presented at the 2016 ASCO Annual Meeting after a 2-years follow-up of the CheckMate-017 and CheckkMate-057 studies show that the 2-year survival rate is higher with nivolumab compared with docetaxel.^38^ Among patients with squamous histology tumours, the 2-year survival rate is 23% with nivolumab versus 8% with docetaxel.^38^ Among patients with non-squamous histology tumours, these values ​​are 29% and 16%, respectively.^38^

A similar effect occurred with pembrolizumab versus docetaxel. After a minimum follow-up of 2 years, the survival rate was 30.1% with pembrolizumab 2 mg/kg, 37.5% with pembrolizumab 10 mg/kg versus 14.5% with docetaxel.^39^

Recently, at the 2017 AACR Annual Meeting, a 5-year follow-up data from the phase I study of nivolumab was released.^40^ According to the authors, the 5-year survival rate was 16%, which is an expressive value compared with the 4% reached with chemotherapy.^40^ The authors assessed the PD-L1 expression of 10 out 16 patients alive after 5 years. 70% of these patients had a PD-L1 expression ≥1%.^40^

In addition, a model of life-time long survival was developed for pembrolizumab versus docetaxel.^41^ The model was based on the risk of disease progression not being constant (the risk of disease progression is higher at the beginning of treatment and decreases progressively).^41^ According to the model, the risk of disease progression is zero after some time (when the tail of the curve is reached).^41^

The initial model based on KeyNote-010 published data found a 5-year survival rate of 25.3%.^41^ Subsequently, the model was repeated based on the prolonged follow-up data previously described and the rate found was 21.5%.^41^ For control purposes, the group of patients treated with docetaxel had a 5-year survival rate estimated at 4.3%, being fully compatible with the expected values ​​classically.^41^

The main criticisms of this model is that it does not represent real follow-up data. In addition, the model used data derived from randomised clinical trial that may not represent the real-life population.

## Immune checkpoint inhibitors in the first-line treatment of NSCLC

The next step was the assessment of immunotherapy in the first-line treatment of NSCLC (table 3). Many of the phase I/II studies cited above included a few patients who were previously untreated, showing promising results, especially for those whose TCs expressed PD-L1.^26–29^ For this reason, all phase III trials enrolled only PD-L1-positive patients to compare immunotherapy as a single agent with a platinum-based regimen for the first-line treatment of NSCLC.^42 43^ The studies showed discrepant results and the PD-L1 positivity threshold used for patient selection was the main difference between CheckMate-026 and KeyNote-024.^42 43^ All patients in both studies were EGFR and ALK wild type.^42 43^

**Table 3:** Data on immunotherapy as single agent for the first-line treatment of NSCLC

	CheckMate-026^42^	KeyNote-024^43^
Immunotherapy vs ICPD	Nivolumab	Pembrolizumab
ORR vs ICPD	26% vs 34%	45% vs 28%
Median PFS vs ICPD	4.2 m vs 5.9 m	10.3 m vs 6.0 m
Median OS vs ICPD	14.4 m vs 13.2 m	NR both arms
HR for OS (95% CI) PD-L1≥5%	1.02 (0.80 to 1.30)	NA
HR for OS (95% CI) PD-L1≥50%	0.90 (0.67 to 1.32)	0.60 (0.41 to 0.89)

ICPD, immune-check points drug; NA, not applicable; NSCLC, non-small cell lung cancer; ORR, objective response rate; OS, overall survival; PFS, progression-free survival.

CheckMate-026^42^ enrolled 541 patients who received nivolumab 3 mg/kg every 2 weeks until disease progression or investigator’s choice of platinum-based doublet (ICPD) chemotherapy every 3 weeks for up to six cycles.^42^ Patients in the ICPD arm who achieved partial response or stable disease after six cycles could be maintained on chemotherapy until disease progression or unacceptable toxicity.^42^

There were more women in the ICPD than in the nivolumab arm (45% vs 32%).^42^ There were no other significant differences in key baseline characteristics between the two groups.^42^ The two most commonly used chemotherapy doublets were pemetrexed/carboplatin (43.7%) and pemetrexed/cisplatin (32.7%) and about 40% of patients received maintenance pemetrexed.^42^ Although the threshold of PD-L1 positivity in CheckMate-026 was ≥1%, the primary end point of the study was the PFS inpatients with PD-L1≥5% (423 patients).^42^

Nivolumab had a worse PFS compared with ICPD (median PFS of 4.2 months vs 5.9 months; (HR 1.15; 95% CI 0.91 to 1.45).^42^ Median OS inpatients with PD-L1 expression ≥5% was 14.4 months with nivolumab and 13.2 months with chemotherapy (HR 1.02; 95% CI 0.80 to 1.30).^42^ The ORR was 26.1% with nivolumab and 33.5% with chemotherapy.^42^ Even among patients with PD-L1 expression ≥50%, a benefit was not observed for nivolumab versus chemotherapy, with a HR for PFS of 1.07 and a HR of 0.90 for OS.^42^

Toxicities in both treatment groups were consistent with previous reports. The most common adverse events with nivolumab were fatigue (21% vs 35.4% with chemotherapy), diarrhoea (13.9% vs 12.9%), decreased appetite (12% vs 27.8%) and nausea (11.6% vs 48.3%).^42^ Grade 3/4 adverse events were uncommon with nivolumab.^42^

In contrast with these findings, KeyNote-024 showed significantly better clinical outcomes for pembrolizumab than chemotherapy inpatients who expressed PD-L1 at a higher threshold of 50% or greater.^43^

Overall, 30.2% of 1653 samples expressed PD-L1 on ≥50% of cells by immunohistochemistry.^43^ Of those who met the PD-L1 expression requirements, 305 were randomised to receive pembrolizumab (154) or ICPD (151), which most commonly included pemetrexed/carboplatin (44%).^43^

The trial achieved its primary end point showing that patients treated with pembrolizumab had a longer PFS than those who received chemotherapy.^43^ The median PFS was 10.3 months with pembrolizumab versus 6 months with chemotherapy (HR 0.50; 95% CI 0.37 to 0.68).^43^ Although the follow-up period was relatively short, 6 month OS rate was 80.2% with pembrolizumab versus 72.4% with chemotherapy (HR 0.60; 95% CI 0.41 to 0.89).^43^ The ORR was also higher with pembrolizumab (45% vs 28%; p=0.0011).^43^

Fewer treatment-related adverse events were seen with pembrolizumab versus chemotherapy (all grades: 73.4% vs 90%; grade 3% to 5 26.6% vs 53.3%).^43^ The most common toxicity with pembrolizumab were diarrhoea (14.3%), fatigue (10.4%) and fever (10.4%).^43^

These results have since changed the management of advanced NSCLC. Patient whose tumours have 50% or greater expression of PD-L1 should now be offered treatment with pembrolizumab and the agent has been approved in a number of jurisdictions.

**Figure 1:**
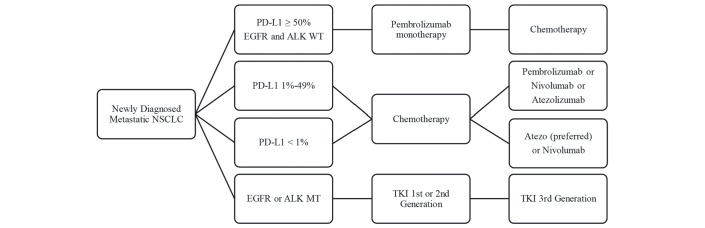
The guideline for the treatment of non-small cell lung cancer (NSCLC) after the development of immunotherapy.

Figure 1 summarises the guideline for the treatment of NSCLC after the development of immunotherapy.

## The role of PD-L1 expression as a biomarker

PD-L1 expression is a logical predictor of responsiveness to PD-1/PD-L1 inhibitors as it is mechanically essential in the immune checkpoint pathway.^21^ Two recent meta-analyses have shown the response to immunotherapy increases proportionally with the extent of PD-L1 expression in TCs.^44–46^ As a general model, the ORR is 2 to 3 times higher for PD-L1-positive patients.^45 46^ Moreover, patients with PD-L1-positive tumours have higher survival rates when treated with ICIs.^45 46^

The indication of immunotherapy as a single agent for the first-line treatment of NSCLC is well established. As cited above, patients must have at least 50% of PD-L1 expression and a wild type EGFR and ALK to be eligible for pembrolizumab. New combinations are under investigation to improve immunogenicity of tumours with PD-L1 <50% and improve the outcomes with immunotherapy. These combinations will be discussed in the next session.

For second-line therapy, ICIs are indicated as single agents regardless of PD-L1 expression (except pembrolizumab that is indicated only for patients with at least 1% of TCs expression). This indication has produced significant discussion regarding the high cost of treatment in this clinical context.^47 48^ The selection of patients eligible for immunotherapy by PD-L1 expression may improve the cost-effectiveness of the treatment and decrease the economic burden of the treatment.^47 48^

Nevertheless, about 10% of patients have some benefit with immunotherapy despite being PD-L1 negative.^31^ Furthermore, the median OS among all PD-L1-negative patients is equal with immunotherapy and with chemotherapy.^31^

Further investigation is required in order to refine PD-L1 expression as a predictive biomarker.

## Further directions for the first-line treatment of PD-L1-negative patients

### Immunotherapy plus chemotherapy

After successful demonstration of efficacy as single-agents, clinical trials are currently assessing PD-1 and PD-L1 inhibitors combined with chemotherapy, other ICs and targeted therapies, in attempts to further improve outcomes for patients with low PD-L1 expression advanced NSCLC.

In an open-label phase II cohort study, 123 patients were randomised to receive pemetrexed/carboplatin (63) or in combination with pembrolizumab(60).^49^ In both groups, chemotherapy was given for four cycles followed by indefinite pemetrexed maintenance while in the investigational arm, pembrolizumab was continued for 24 months.^49^

The primary end point for this study was achieved, with an improvement in ORR.^49^ The ORR was 55% with pembrolizumab plus chemotherapy compared with 29% for chemotherapy alone (p=0.0016).^49^ The risk of progression was also reduced by 47% with pembrolizumab (median PFS 13 months vs 8.9 months; p=0.0102).^49^ The follow-up was insufficient to evaluate the OS (median not reached in both arms).^49^ In assessments of PD-L1 staining, patients with PD-L1 <1% had an ORR of 57% with pembrolizumab combination compared with 13% in the chemotherapy arm.^49^

Pembrolizumab was well tolerated.^49^ The most frequent all-grade treatment-related adverse events in the pembrolizumab combination and chemotherapy isolated arms, respectively, were fatigue (64% vs 40%), nausea (58% vs 44%), anaemia (32% vs 53%), vomiting (25% vs 18%) and diarrhoea (20% vs 10%).^49^ Adverse events led to treatment discontinuations for 10% of patients in the pembrolizumab arm compared with 13% in the control group.^49^

Early findings for chemotherapy combined with PD-1/PD-L1 inhibitors showed promising signs of efficacy, resulting in the initiation of several phase III trials and the recent approval of this combination for use in the USA by Food and Drug Administration. Nevertheless, combination strategies have some limitations. The first is the need to confirm putative benefits in a phase III trial. The second one is the high cost of aggregating treatment with immunotherapy and chemotherapy.

### Immunotherapy combinations

In a large phase I study, a single arm assessed the combination of nivolumab with ipilimumab (an immune checkpoint inhibitor that stimulate lymphocyte activity by binding of CTLA-4 receptor).^50^ In a report of this study, 77 chemotherapy-naive patients received ipilimumab every 6 weeks or every 12 weeks plus nivolumab every 2 weeks until disease progression or unacceptable toxicity.^50^Thirty-three patients (43%) achieved a partial response and the median PFS was 8.1 months for the nivolumab 3 mg/kg every 2 weeks plus ipilimumab 1 mg/kg each 12 weeks arm and 3.9 months for the nivolumab 3 mg/kg every 2 weeks plus ipilimumab 1 mg/kg every 6 weeks.^50^ The median OS was not reached in both arms.^50^ The combination was effective regardless of PD-L1 expression.^50^

Unfortunately, the majority of patients (82%) had some adverse event and one third had a severe adverse event.^50^ The most common severe immune-related adverse events were diarrhoea (5%), colitis (5%) and pneumonitis (4%).^50^

Another phase Ib trial assessed the combination of a PD-L1 inhibitor (durvalumab) with a CTLA-4 inhibitor (tremelimumab).^51^ This study enrolled 102 patients with immunotherapy-naive (6% was also chemotherapy-naive) for treatment.^51^

The ORR was 23% in the combined tremelimumab 1 mg/kg cohort and the benefit was independent of PD-L1 expression.^51^ Two of nine patients with PD-L1-positive tumours and 4 of 10 patients with no PD-L1 staining achieved objective response.^51^ The study did not assess PFS and OS.^51^

Once again, the safety of this combination was the major concern. The most frequent treatment-related grade 3 and 4 adverse events were diarrhoea (11%), colitis (9%) and increased lipase (8%).^51^ Discontinuations attributable to toxicity occurred in 29 of 102 patients.^51^ Three deaths were related to treatment.^51^

The combination of CTLA-4 inhibitors with PD-1/PD-L1 inhibitors has significant limitations showing incorporation into the clinical practice. The main concern is the very high cost of these combinations. The clinical outcomes of these combinations must be significantly better than standard therapy to achieve economy. The other limitation is the harmful toxicity profile of this treatment. The adverse events observed are worse than expected with single agent immunotherapy. This may be a result of a toxic synergism between these ICIs.

The combination of anti-PD1/anti-PDL1 with other ICIs has several limitations. Translating theoretical and in vitro synergy into in vivo synergy and subsequent clinical benefit for patients is challenging. Another limitation is the very high cost of these combinations. Finally, the toxicity profile of these combinations seems to be more harmful than expected (such as observed with anti-CTLA4 plus anti-PD1/anti-PDL1).

### Vaccines

In NSCLC context, vaccine therapy may be divided into TC vaccines (autologous or allogeneic TCs), and antigen-based vaccines. In general vaccines are administered with adjuvants, whose purpose is to stimulate the immune response without have intrinsic antigen effect.^52 53^ Although vaccines have been evaluated for the treatment of NSCLC at different stages, they almost all failed to demonstrate any benefit.

- *Vaccines as maintenance therapy:*

MUC1 is overexpressed and aberrantly glycosylated in NSCLC, making it a target for immunotherapy.^54^ A phase IIB study randomised 171 patients with advanced NSCLC without disease progression after first-line chemotherapy to receive tecemotide (a peptide vaccine targeting MUC1) or best supportive care alone.^54^ The median OS was higher in the vaccine group, however, this was not statistically significant (17.4 months vs 13 months; HR 0.75; 95% CI 0.53 to 1.04).^54^

Lucanix (belagenpumatucel-L) is a tumour vaccine, which is a compound of four allogeneic NSCLC cell lines modified with transforming growth factor-b2-antisense plasmid.^55^ A phase II trial randomised 532 patients with advanced NSCLC without progression after platinum-based chemotherapy to maintenance belagenpumatucel-L or placebo.^55^ Although the vaccine was well tolerated, there was not any benefit with this treatment.^55^ The median PFS was 4.3 months with vaccine versus 4 months with placebo.^55^ The median OS was 20.3 months with vaccine versus 17.8 months with placebo (HR 0.94; 95% CI 0.73 to 1.20).^55^

Racotumomab-alum is a tumour vaccine targeting the NeuGGM3 tumour-associated ganglioside.^56^ It was assessed in a phase II study, which randomised 176 patients with advanced NSCLC who achieved at least stable disease after first-line chemotherapy to receive racotumomab-alum or placebo.^56^ The vaccine improved not only the median PFS (5.3 months vs 3.9 months; HR 0.73; 95% CI 0.53 to 0.99), but also the median OS (8.2 months vs 6.8 months; HR 0.63; 95% CI 0.46 to 0.87) with racotumomab-alum group compared with placebo.^56^

Vx-001 is an HLA-A*0201-restricted vaccine targeting the human telomerase reverse transcriptase tumour antigen.^57^ Patients with positive HLA-A*0201 tumour antigen and residual or progressive disease after first-line chemotherapy were treated by six doses of Vx-001 in a phase II trial.^57^ The ORR and stable disease rate were 7% and 28%, respectively.^57^ The median PFS was 3.8 months and the median OS was 19 months.^57^ In a subset analysis, patients who mounted immune responses (defined by number of interferon-g-spots-forming-cells from blood mononuclear cells significantly increasing after vaccination compared with the background) had prolonged median OS compared with patients who did not mount a response (40 months vs 9.2 months; p=0.02).^57^ The most common adverse events were nausea, fatigue, anaemia and injection-site reaction.^57^

- *Vaccines as first-line treatment*

The TG4010 vaccine (modified vaccine virus Ankara containing the sequence for interleukin-2 and MUC-1) has a tumour-specific antigen sequence which is overexpressed in various epithelial tumours, such as lung cancer.^58^ A phase IIb/III trial compared the addition of TG4010 immunotherapy to chemotherapy with placebo plus chemotherapy inpatients with advanced NSCLC and MUC1 expression ≥50%.^58^ The study randomised 222 patients to receive TG4010 versus placebo, in addition to platinum doublet-based chemotherapy.^58^ The primary endpoint of improvement in PFS was achieved (median PFS 5.9 months vs 5.1 months; HR 0.74; 95% CI 0.55 to 0.98).^58^ Otherwise, the median OS was also superior for TG4010 compared with placebo (12.7 months vs 10.6 months; HR 0.78; 95% CI 0.57 to 1.06).^58^ Most common adverse events were grade 1 to 2 injection-site reactions.^58^

- *Vaccines in previously treated patients*

A phase II study has evaluated a tumour vaccine whose composition is a granulocyte-macrophage colony-stimulating factor–producing and CD40L-expressing bystander cell line and allogeneic TCs.^59^ Twenty-four patients were enrolled with a median of four previous lines of systemic treatment.^59^ There was no objective response, the median PFS was 1.7 months and the median OS was 7.9 months.^59^ The most common adverse events were mild headache and injection site reaction.^59^

GVAX is a tumour vaccine consisting of autologous TCs mixed with an allogeneic cell line secreting granulocyte-macrophage colony-stimulating factor studied in a phase I/II trial.^60^ Eighty-six patients with advanced NSCLC had tumour harvested to vaccine preparation and 49 patients received the vaccine treatment.^60^ There was no objective responses, the median PFS was 4.4 months and the median OS was 7 months.^60^ Common adverse events included mild injection site reactions, fatigue, dyspnoea, nausea and fever.^60^

### Adaptive T-cell therapy

Adaptive T-cell therapy is the reinfusion back into the patient of expanded populations of tumour-infiltrating lymphocytes (TILs) collected from tumour resection specimens. Some patients with melanoma had response with this therapy,^61–63^ although this strategy may be harder for most other solid tumour types due to difficulty in identification and ex vivo culturing of TILs.

Consequently, an alternative strategy has been developed, involving genetically modifying otherwise tumour non-reactive T cells to bear tumour reactivity. CD8+ and CD4+ T cells are collected via leukapharesis, and expanded via coculture with artificial antigen-presenting cells. The final result is a T cell expressing either a T cell receptor (TCR) clone or a chimeric antigen receptor (CAR), specific to a tumour-associated antigen (TAA).^64^

TAAs that have been targeted via engineered T cells in lung cancer include: (1) NYESO1 (NCT01697527, NCT01967823), a cancer testis antigen found in up to 30% of lung cancers^65^ (2) VEGF receptor 2 (NCT01218867), expressed in the majority of lung cancer samples^66^ (3) MAGE-A3 (NCT02111850), found over 40% of lung cancers.^67^

Although enthusiastic results inpatients with synovial cell sarcoma, melanoma and leukaemia, Engineered T-cell therapy has not demonstrated efficacy in solid tumours.^64 68 69^ The only exception is CAR-engineered T cells that showed partial responses in a small proportion of patients with mesothelioma.^70^

There are several aspects that inhibits adoptive T-cell therapy efficacy: heterogeneous TAA expression and TAA shedding, short T-cell survival/persistence, suboptimal T-cell trafficking, the barrier of tumour-associated stroma, the presence of suppressive immune cells, upregulation of inhibitory checkpoints, the expression of regulatory genes, lack of oxygen and cellular nutrients and immunosuppressive soluble factors.^64^

There are many problems to solve, based on tumour and T-cell microenvironments, to develop adoptive T-cell therapy into a treatment for lung cancer.

**Figure 2:**
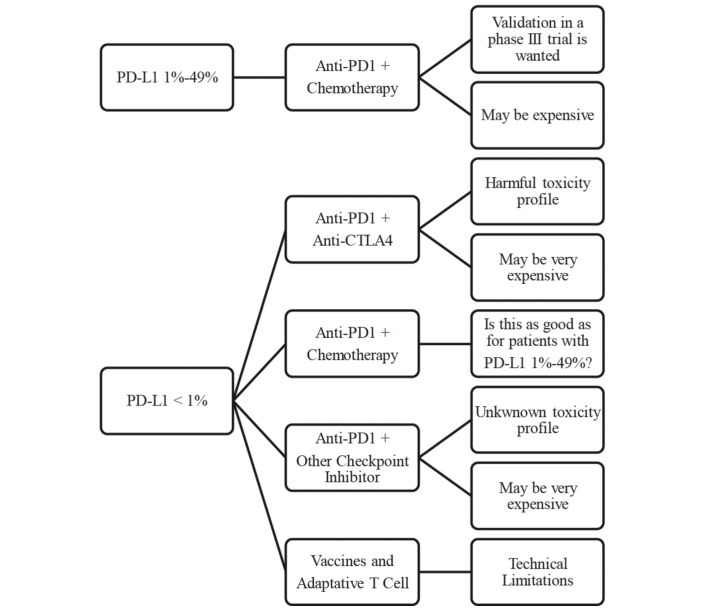
Hypothetical options for the first-line treatment of non-small cell lung cancer for patients with programmed death receptor ligand-1 (PD-L1)<50%.

Figure 2 shows hypothetical options for the first-line treatment of NSCLC for patients with PD2L1< 50%.

## Conclusion

Immunotherapy has changed the treatment of NSCLC. While it has become a second-line therapy of choice, recent clinical trial data is shifting the paradigm in the first-line treatment as well, for a limited proportion of patients (30% with PD-L1≥50%). Further strategies are under development in order to trump traditional chemotherapy and extend the benefits to a higher proportion of patients.
